# Patient presentation and physician management of upper respiratory tract infections: a retrospective review of over 5 million primary clinic consultations in Hong Kong

**DOI:** 10.1186/1471-2296-15-95

**Published:** 2014-05-13

**Authors:** Kenny Kung, Carmen Ka Man Wong, Samuel Yeung Shan Wong, Augustine Lam, Christy Ka Yan Chan, Sian Griffiths, Chris Butler

**Affiliations:** 1 Division of Family Medicine, School of Public Health & Primary Care, Chinese University of Hong Kong, Shatin, Hong Kong; 2 Department of Family Medicine, the Hospital Authority, Kowloon, Hong Kong; 3 Department of General Practice, Cardiff University, Cardiff, UK; 4 Jockey School of Public Health, Prince of Wales Hospital, Room 408, 32 Ngan Shing Street, Shatin, NT, Hong Kong

**Keywords:** Upper respiratory tract infection, Primary care, Pharmacology

## Abstract

**Background:**

Upper respiratory tract infection (URTI) has a significant healthcare burden worldwide. Considerable resources are consumed through health care consultations and prescribed treatment, despite evidence for little or no effect on recovery. Patterns of consultations and care including use of symptomatic medications and antibiotics for upper respiratory tract infections are poorly described.

**Methods:**

We performed a retrospective review of computerized clinical data on patients presenting to all public primary care clinics in Hong Kong with symptoms of respiratory tract infections. International Classification of Primary care (ICPC)codes used to identify patients included otitis media (H71), streptococcal pharyngitis (R72), acute URTI (R74), acute sinusitis (R75), acute tonsillitis (R76), acute laryngitis (R77), and influenza (R80). Sociodemographic variables such as gender, age, chronic illness status, attendance date, type and duration of drug prescribed were also collected.

**Results:**

Of the 5,529,755 primary care consultations for respiratory symptoms from 2005 to 2010, 98% resulted in a prescription. Prescription patterns of symptomatic medication were largely similar across the 5 years. In 2010 the mean number of drugs prescribed per consultation was 3.2, of which the commonly prescribed medication were sedating antihistamines (79.9%), analgesia (58.9%), throat lozenges (40.4%) and expectorant cough syrup (33.8%). During the study period, there was an overall decline in antibiotic prescription (8.1% to 5.1%). However, in consultations where the given diagnosis was otitis media (H71), streptococcal pharyngitis (R72), acute sinusitis (R75) or acute laryngitis (R76), over 90% resulted in antibiotic prescription.

**Conclusion:**

There was a decline in overall antibiotic prescription over the study period. However, the use of antibiotics was high in some conditions e.g. otitis media and acute laryngitis a. Multiple symptomatic medications were given for upper respiratory tract infections. Further research is needed to develop clinical and patients directed interventions to reduce the number of prescriptions of symptomatic medications and antibiotics that could reduce costs for health care services and iatrogenic risk to patients.

## Background

Respiratory infections are the commonest health problem encountered in primary care worldwide [1-3], amounting to between 16% to over 60% of attendances depending on locality [4,5]. Upper respiratory tract infection (URTI) is an important cause of reduced activity days, school and work loss, impaired school performance, and increased healthcare utilization [6], resulting in substantial economic burden.

There is little data on actual prescribing practice for URTI in Asia. Evidence shows that symptomatic medications including antibiotics are of marginal or no benefit [7-13]. However, there are widespread variations in URTI management. A telephone survey of Auckland GPs found that 95% would, on occasions, prescribe as-needed medications (instructions for patient to fill prescriptions if condition worsens), including antibiotics [14]. In a European study, the number of medication types per patient varied from 0.82 to 3.55 per patient per year [15]. Interestingly, the antibiotic prescription rate in this European study did not vary with the overall medication prescription rate. A study in Zimbabwe highlighted widespread non-evidence based prescribing for URTI patients [16]. The actual prescribing practice among Chinese primary care physicians for URTI symptoms has not previously been investigated, although anecdotal observations show that medications for symptomatic relief are commonly used. Understanding the characteristics of patients who consult with URTI symptoms and the prescribing practices of primary care clinicians could identify opportunities for intervention such as reducing unnecessary prescriptions of antibiotic and symptomatic medication and reallocating resources for other medical conditions in primary care such as chronic disease management.

We therefore reviewed the characteristics of patients attending Hong Kong’s public primary care clinics (PPCCs) with URTI symptoms, and the types of medications clinicians prescribed for URTI symptoms.

## Methods

We performed a retrospective review of computerized clinical data on patients presenting to PPCCs with upper respiratory tract symptoms between 1^st^ January 2005 and 31^st^ December 2010. Upper respiratory tract symptoms were defined by specific ICPC (International Classification of Primary Care) codes agreed upon by an expert panel of local primary care physicians including:

• H71 – otitis media

• R72 – streptococcal pharyngitis

• R74 – acute upper respiratory tract infection

• R75 – acute sinusitis

• R76 – acute tonsillitis

• R77 – acute laryngitis

• R80 – influenza

There are 74 PPCCs in Hong Kong covering the entire population 7 million, with over 5 million attendances annually [17]. Consultations fees are subsidized for permanent residents (patient pays USD5.8 per consultation, inclusive of medications and investigations), while specific populations (government workers and those on financial assistance) receive free consultations. Existing literature suggests that less than 100,000 attendances are made to local emergency departments for non-urgent respiratory tract infections annually [18,19], while 26% of private sector consultations (accounting for 50% of Hong Kong’s primary care) are related to respiratory tract infections [1].

Data on gender, age, chronic illness status, attendance date, type and duration of drug prescribed for URTI were extracted from the system-wide electronic record system (Clinical Data Analysis & Reporting System [CDARS]). There is no information from CDARS for patients’ financial status; nevertheless, those requiring financial assistance from the government can be identified separately from those paying the usual fee. Previous publications using CDARS have demonstrated the accuracy and completeness of the data retrieved from this system [20].

We defined a repeat attendance for the same URTI episode (which we considered as the same illness episode) as any attendances for URTI symptoms within 28 days of the previous URTI attendance. Medications were grouped into specific drug classes, including analgesia, antibiotics, antihistamines, cough syrup, mucolytic, s α-agonists, β-agonists, theophylline and other including throat lozenges, lysozyme.

All statistical analysis was performed using SPSS version 18.0 for Windows. Descriptive statistics were calculated using mean and range. Chi-square test was used to compare categorical variables across the different years. All p values <0.05 were regarded as statistically significant.

This study was approved by the Clinical Research Ethics Committee of the Chinese University of Hong Kong and New Territories East Cluster of the Hospital Authority.

## Results

There were 5,529,755 attendances for RTIs from 2005 to 2010. Patient demographics and the conditions diagnosed are summarized in Table 1.

**Table 1:** Patient demographics and diagnosed conditions

	**Year of attendance**					
	**2005**	**2006**	**2007**	**2008**	**2009**	**2010**
**Annual attendance for all respiratory infections**	997768	937686	923504	966739	813874	895978
**Distribution of annual attendances number (number of patients)**						
**1 attendance**	59.9% (319154)	59.7% (296239)	57.5% (269327)	56.7% (274308)	59.4% (253995)	57.8% (264110)
**2-4 attendances**	34.2% (181992)	34.0% (168824)	35.3% (165316)	35.9% (173343)	34.3% (146461)	35.3% (161307)
**5-8 attendances**	4.9% (26157)	5.2% (25765)	5.8% (27347)	6.1% (29305)	5.2% (22411)	5.8% (26308)
**>8 attendances**	1.0% (5495)	1.1% (5359)	1.3% (6034)	1.3% (6517)	1.1% (4735)	1.2% (5549)
**Patient headcount**	532798	496187	468024	483473	427602	457274
**Mean attendance per patient per year**	1.9	1.93	2.0	2.0	1.9	2.0
**Mean age (years)**	41.1	41.2	42.9	43.8	44.3	45.6
**Gender**						
**Female**	56.4%	56.1%	56.9%	57.0%	56.4%	57.0%
**On government subsidy**	20.7%	20.4%	19.5%	21.0%	21.4%	21.0%
**Chronic illness**						
**Ischaemic heart disease**	1.6% (8440)	1.5% (7578)	1.5% (7219)	1.6% (7594)	1.5% (6324)	1.5% (6729)
**Hypertension**	22.8% (121304)	21.8% (108344)	23.2% (108397)	23.9% (115461)	23.9% (102066)	24.0% (109558)
**Stroke**	1.6% (8290)	1.4% (7191)	1.5% (7151)	1.6% (7580)	1.4% (6128)	1.4% (6392)
**Prostatism**	2.6% (13905)	2.7% (13160)	2.8% (13219)	2.9% (14072)	2.9% (12610)	2.9% (13374)
**Asthma**	2.9% (15264)	3.0% (14904)	3.2% (14866)	3.1% (15146)	3.2% (13563)	2.9% (13358)
**COPD**	1.5% (7797)	1.4% (7191)	1.4% (6679)	1.3% (6458)	1.3% (5595)	1.1% (5197)
**Coded conditions**						
**H71 (otitis media)**	0.29%	0.34%	0.31%	0.33%	0.33%	0.33%
**R72 (streptococcal**	0.02%	0.03%	0.02%	0.03%	0.04%	0.04%
**pharyngitis)**	97.35%	96.99%	97.07%	96.30%	95.42%	94.25%
**R74 (acute URTI)**	0.15%	0.17%	0.20%	0.21%	0.26%	0.25%
**R75 (acute sinusitis)**	0.49%	0.53%	0.44%	0.67%	0.88%	1.02%
**R76 (acute tonsillitis)**	0.04%	0.04%	0.03%	0.03%	0.04%	0.07%
**R77 (acute laryngitis)**	0.26%	0.33%	0.29%	0.27%	0.69%	0.97%
**R80 (influenza)**						

### Patient presentation

There were 5,529,755 consultations for URTI symptoms in the study period for the patient population of 1,181,816 [21]. The distribution of annual attendances was similar across the six years with the majority (approx 60%) of patients presenting once for symptoms of upper respiratory tract infections. Over a third of the patients (approximately 35%) attended 2-4 times per year. There was a 16% drop in attendances in 2009. Overall, there was a significant increase (p < 0.001) in the mean number of annual attendances per patient.

The mean age of presentation shows a steady increase from 41.1 to 45.6 years from 2005 -2010. Patients were more likely to be female (approx 57%) and on average one fifth of those presenting were on government subsidy. Over 94% were coded as upper respiratory tract infection (R74). Over the 6 years, there was a significant increase (p < 0.001) in the number of patients coded with non-R74 conditions.

### Prescription of medication

Of the 5,529,755 primary care consultations for URTI symptoms from 2005 to 2010, 98% resulted in a prescription of at least one medication (Table 2). Prescription patterns of symptomatic medication were largely similar across the 5 years, but there was a small but significant rise in the number of drugs given per consultation (p < 0.001). The most commonly prescribed medications were sedating antihistamines (79.9%), analgesia (58.9%), throat lozenges (40.4%) and expectorant cough syrup (33.8%). During the study period, there was a significant decline in antibiotic prescription from 8.1% to 5.1% (p < 0.001). However, in consultations, where the given diagnosis was otitis media (H71), streptococcal pharyngitis (R72), acute sinusitis (R75) and acute laryngitis (R76), over 90% resulted in antibiotic prescription (Table 3). Almost 50% of patients diagnosed with influenza were prescribed antibiotics. The proportion was much less among those coded with R74 (acute URTI).

**Table 2:** Details on drugs prescribed

	**Year of attendance**						
	**2005**	**2006**	**2007**	**2008**	**2009**	**2010**	
**Mean number of drugs**	3.1	3.1	3.2	3.2	3.2	3.2	
**Number of drug items per prescription**							
**0**	2.7%	2.7%	2.3%	1.3%	1.3%	1.1%	
**1**	5.7%	5.5%	5.1%	5.1%	5.5%	5.0%	
**2**	21.2%	20.4%	19.6%	19.7%	19.8%	18.3%	
**3**	34.6%	33.8%	33.3%	33.0%	32.4%	32.1%	
**4**	27.8%	28.5%	29.7%	30.9%	31.0%	33.0%	
**>4**	8.1%	9.2%	10.0%	10.0%	10.0%	10.5%	
**Proportion of prescriptions containing**							** Current evidence**
**Analgesics**							
**Paracetamol**	58.3%	60.1%	59.8%	59.1%	58.7%	58.9%	May reduce fever
**NSAIDs**	3.0%	3.6%	4.2%	4.3%	4.5%	4.9%	May reduce pain
**COX-II inhibitors**	0.0%	0.0%	0.0%	0.0%	0.0%	0.0%	No evidence
**Antibiotics**	8.1%	8.1%	6.8%	4.9%	4.4%	5.1%	
**Antihistamines**							
**Sedating**	81.1%	80.1%	82.3%	82.4%	80.8%	79.9%	Ineffective
**Non-sedating**	0.5%	0.3%	0.3%	0.3%	0.6%	1.2%	
**Cough syrup**							
**Expectorant**	36.8%	35.6%	36.3%	36.3%	34.0%	33.8%	Not recommended in children
**Codeine**	7.1%	6.9%	6.7%	6.5%	6.6%	6.3%	Limited evidence
**Mucolytic**	20.9%	22.2%	23.0%	24.0%	24.0%	25.0%	May reduce symptoms
**Oral α-agonist**	0.6%	0.4%	0.3%	0.2%	0.2%	0.2%	Modest effect in adults
**Oral β-agonist**	4.4%	4.7%	4.2%	4.1%	3.7%	4.1%	No evidence
**Theophylline**	1.1%	1.0%	1.0%	0.9%	0.8%	0.9%	No evidence
**Others**							
**Throat lozenges**	34.7%	34.9%	35.6%	37.5%	39.9%	40.4%	No evidence
**Lysozyme**	10.6%	11.8%	13.8%	14.5%	14.9%	15.6%	No evidence
**Neozep**	0.6%	0.3%	0.3%	0.2%	0.0%	0.1%	

**Table 3:** Proportion of antibiotic prescribed in different URTIs

	**Condition**						
**Year**	**H71**	**R72**	**R74**	**R75**	**R76**	**R77**	**R80**
**2005**	94.4% (2816)	89.2% (213)	6.5% (957615)	90.7% (1523)	95.2% (4776)	23.9% (348)	54.0% (2556)
**2006**	95.4% (3135)	80.6% (294)	6.3% (897381)	86.1% (1529)	96.0% (4915)	25.4% (378)	52.8% (3087)
**2007**	95.2% (2798)	81.7% (164)	5.1% (889038)	85.0% (1788)	94.7% (4005)	26.9% (283)	50.6% (2669)
**2008**	94.1% (3179)	93.9% (245)	2.6% (937252)	91.6% (2024)	96.7% (6543)	59.1% (281)	47.0% (2623)
**2009**	94.4% (2683)	95.5% (352)	1.8% (782692)	92.0% (2138)	97.8% (7233)	68.6% (338)	43.1% (5697)
**2010**	94.4% (3044)	97.8% (320)	1.9% (858979)	91.3% (2317)	97.9% (9317)	73.6% (658)	48.2% (8882)

Bracketed values indicate the number of attendances where the specified ICPC code was assigned.

## Discussion

### Main study findings

Consultations for URTI symptoms account for almost 20% of attendances per year in Hong Kong’s public primary care (around 900,000 out of 5 million attendances annually [17]). There was a drop in attendances in 2009, which coincides with the swine flu outbreak that year, where patients with influenza like illness were advised to go to designated fever clinics. There is an increasing mean age of patients presenting to primary care for upper respiratory tract infections which may reflect the increasing age of the population and those presenting to primary care. 98% of consultations were associated with a prescription, which on average included four drug classes. Antibiotics were prescribed in 5% of consultations.

### Antibiotic prescribing

The overall antibiotic prescription rate observed in this study was much lower than in other countries [22-25]. Although patients with uncomplicated URTIs (R74) comprise the large majority of the population, it appears that the overall volume of antibiotics prescribed remains low. However, nearly all those coded with H71 (otitis media), R72 (streptococcal pharyngitis), R75 (acute sinusitis) and R76 (acute tonsillitis) were almost always prescribed antibiotics, with an increasing trend among R72 and R77. Furthermore, around 50% of those diagnosed with influenza (R80) were also prescribed with antibiotics. Prescribing pressure [26] from patients may be an issue, especially among those with more troubling symptoms. This could also reflect clinicians’ 'coding creep’ or 'diagnostic drift’ with the tendency to use non-R74 codes in consultations where antibiotics were prescribed. Further education and training in terms of diagnosis, management and use of correct coding may need to be instated. Whilst financial incentive is an important contributory factor for 'coding creep’ in some countries [27-29], this is not an issue in Hong Kong where there is no pay-for-performance system in place. Further education of doctors in the appropriate management of otitis media, streptococcal pharyngitic, acute sinusitis and acute tonsillitis may help to reduce unnecessary antibiotic prescription.

### Symptomatic drug utilization

At least 3 medications are prescribed for each URTI episode. This is higher than the URTI drug prescription patterns of Dutch doctors, where a mean of 0.8 drugs were prescribed per patient. Possible explanations for a higher prescription number may be related to the nature of the Hong Kong healthcare system, where consultation fees in PPCCs are heavily subsidized and includes the cost of medication, patients’ relatively low self medication rate [30] and high expectations for receiving a prescription for medication during consultations [31]. The slight increase in the number of medications prescribed coincided with an increase in annual attendances per patient for URTI, suggesting that this prescribing habit may be encouraging patients to consult. Although one previous European study indicated that medication prescribing rates did not vary with antibiotic prescription rate [15], further research would be warranted in view of the overall low antibiotic prescription volume.

A large variety of symptomatic medications were prescribed, including α and β-agonists, lysozyme and mucolytics. There is little empirical evidence that these medicines are effective for URTI [32-35]. Anecdotally, it is not uncommon for patients to request a medication for each symptom they experience, which may help explain why patients are prescribed antitussives, antihistamines, mucolytics and throat lozenges. Among these prescriptions, 80% were on sedating antihistamines; 6% were given opioid containing medications; and a minority was prescribed theophyllines, α-agonists or β-agonists. There is a need for further research on the expectations of symptomatic medication prescriptions as recipients of these medications included children, and patients with chronic disease in which medication side effects could be detrimental e.g. cardiac diseases, prostatic symptoms or respiratory problems [36-39].

### Implication for health service provision and future research

Compared with European countries [40-43], Hong Kong’s public primary care appears to exhibit low antibiotic prescribing behaviour for symptoms of upper respiratory tract infections. On the other hand, the prescription rate for non-antibiotic medications is exceptionally high. It may be that prescription of symptomatic medication has managed to displace the expectation of antibiotic prescription. However, symptomatic drug prescription is high, and is estimated to cost the public healthcare system over 1.2 million US dollars per year. Other costs including PPCCs’ facilities and human resources, as well as the societal impact of health services utilization for URTI have not been included.

Previous studies have shown that a significant proportion of primary care physicians believe patients should be empowered to self manage uncomplicated URTI [44]. Nevertheless, many patients continue to consult doctors within the first two days of their illnesses [30,31] despite its self-limiting nature. Public health initiatives are therefore needed in order to promote self-management and appropriate indications for help seeking. The gap between clinical evidence for pharmacological intervention, doctors’ prescribing behavior and patient expectation must also be addressed in order to safeguard patient wellbeing and the appropriate use of health care resources. Primary care physicians can play an active role to empower patients to self-manage symptoms and in reducing the reliance on unnecessary medications whenever appropriate. Further studies looking into cultural differences in expectations and management of URTI like symptoms can better inform effective regional educational interventions.

### Strengths and limitations of this study

This is the first study of patient characteristics and prescribed medication among patients presenting to public primary care clinics with URTIs in the South China Region. The availability of electronic patient data from all PPCCs in Hong Kong ensured coverage of all patients in the public primary care setting, allowing an accurate analysis into the health services utilization related to URTIs. Internal audits within the Hospital Authority (unpublished data) have indicated that the ICPC coding rate among PPCCs is generally over 90%. Two studies examining ICPC coding accuracy over the recent few years [45,46] have shown that 96% to 98% of cases are coded accurately, especially if the case is not complex. These imply that the data obtained for this study accurately reflects the public primary care situation in our locality.

Those who utilize public clinics in primary care are more likely to be of lower socio-economic status, elderly and individuals with chronic conditions [47], this is reflected in our study where one fifth is on financial assistance and the increasing age of presentation. However, results do not include consultations in the private sector which is likely to see patients who are more affluent and younger. Meanwhile this study has important implications as older people are more prone to drug-drug interactions and adverse events from the prescriptions of medications for URTI. Moreover, as there is a high rate of 'doctor shopping’ among patients in primary care in Hong Kong, this pattern of high utilization of symptomatic prescriptions for URTI in Hong Kong is likely to be common among most patients consulting primary care in Hong Kong, both in the private sector and in government run clinics given similar expectations.

## Conclusions

Overall the rate of antibiotic prescription for patients attending Hong Kong’s public primary care is low, although the rate is high among those diagnosed with otitis media, pharyngitis, sinusitis and tonsillitis. On the other hand, the number symptomatic medications prescribed is high, and its relationship with increasing doctor consultations and low overall antibiotic prescription need to be further explored. Gaps have been identified between primary care practice and evidence based management of upper respiratory tract infections. There is a huge potential to save resources on inappropriate antibiotic use, unnecessary prescription of symptomatic medication and consultation appointments through doctor and patient education.

## Competing interests

The authors declare that they have no competing interests.

## Author contributions

KK/CW conceptualised the whole study. KK researched data and wrote the manuscript, with further analysis performed by CKYC. CW/SYSW/AL/SG/CCB reviewed the manuscript. CCB contributed to discussion and reviewed the manuscript. All authors declare that there are no financial relationships with any organisations that might have an interest in the submitted work, and that no presentations have been made for this research. All authors read and approved the final manuscript.

## Pre-publication history

The pre-publication history for this paper can be accessed here:

http://www.biomedcentral.com/1471-2296/15/95/prepub
